# Long-term Outcomes of Treat and Extend Regimen of Anti-vascular Endothelial Growth Factor in Neovascular Age-related Macular Degeneration

**DOI:** 10.18502/jovr.v15i3.7452

**Published:** 2020-07-29

**Authors:** Andy Lee, Pooja G Garg, Alice T Lyon, Rukhsana Mirza, Manjot K Gill

**Affiliations:** ^1^Department of Ophthalmology, Northwestern University Feinberg School of Medicine, Chicago, IL, USA

**Keywords:** Age-related Macular Degeneration (AMD), Intraocular Drugs, Visual Acuity

## Abstract

**Purpose:**

This study describes the long-term visual and anatomic outcomes of anti-vascular endothelial growth factor (VEGF) treatment using a treat and extend dosing regimen.

**Methods:**

This cross-sectional cohort study consisted of 224 treatment-naïve eyes with neovascular age-related macular degeneration (NV-AMD) from 202 patients that were treated with anti-VEGF agents bevacizumab, ranibizumab, and aflibercept using a treat and extend (TAE) regimen by four physician investigators in a large urban referral center from 2008 to 2015. Subjects were evaluated for visual acuity, injection frequency, and optical coherence tomography (OCT).

**Results:**

Over a seven-year follow-up period (mean 3.4 years), an average 20.2 ± 14.7 injections were administered with 8.4 injections in the first year and 5.5 injections by the seventh year of remaining eyes undergoing treatment. Visual acuity was 0.70 logMAR (20/100 Snellen) at the first visit and 0.67 logMAR (20/93 Snellen) at the final visit, with 74% of eyes maintaining or gaining more than 2 lines of vision. Long-term, 45.1% of eyes achieved 20/50 or better, while 27.1% were 20/200 or worse. Of the treated patients, 61.2% received monotherapy with no difference in visual acuity outcomes or number of injections between the agents used. OCT analysis showed decreased fluid from initial to final follow-up visit: 70.1–15.6% with sub-retinal fluid (SRF) and 47.3–18.8% with intra-retinal fluid (IRF) with no difference between the agents were used.

**Conclusion:**

This study demonstrates that most patients (74%) improve or maintain visual acuity long-term using a TAE model with a significant portion (45.1%) achieving 20/50 or better visual acuity with sustained treatment.

##  INTRODUCTION

Neovascular age-related macular degeneration (NV-AMD) is the leading cause of vision loss in individuals aged 50 years or older. Over the past decade, treatment has evolved to control subfoveal choroidal neovascularization (CNV) growth with intravitreal drug delivery directed toward inhibition of vascular endothelial growth factor (VEGF). Specifically, the MARINA and ANCHOR studies were amongst the first to demonstrate the effects of targeting angiogenesis by blocking VEGF-A with ranibizumab, a recombinant humanized monoclonal antibody fragment (Fab). These studies clearly showed that monthly treatment was beneficial in preventing vision loss and allowing for visual gain compared to sham and photodynamic therapy (PDT), respectively, over a two-year period.^[1,2,3]^ The VIEW 1 and 2 trials demonstrated the efficacy of aflibercept, a soluble decoy receptor fusion protein with a higher affinity to VEGF-A and VEGF-B as well as placental growth factor (PIGF) with decreased treatment burden allowing improvement or maintenance of vision over two years.^[4,5]^ However, monthly or bimonthly injections along with monthly follow-up is challenging for patients to maintain in clinical practice.

Due to treatment burden, pro re nata (PRN) treatment was studied to examine the effects of monthly follow-up with an individualized retreatment regimen. CATT and IVAN trials demonstrated equivalent efficacy between ranibizumab versus bevacizumab; however, there was an overall less favorable outcome in the PRN arms compared to monthly dosing with respect to final visual acuity.^[6,7,8]^

In 2009, Freund and colleagues were the first to describe the “treat-and-extend (TAE)” regimen with treatment of Type 3 CNV lesions in a small cohort over three years.^[9]^ With use of ranibizumab and/or bevacizumab, they showed an overall improvement of vision from 20/80 to 20/40 with an average of 6–7 injections per year. Since then, several other retrospective studies have proposed using a TAE approach as an alternate to monthly or PRN dosing to reduce treatment burden while maintaining or improving visual outcome.^[10,11,12]^

Despite the multitude of trials demonstrating the safety and efficacy of anti-VEGF drug therapy, there have been limited studies describing the long-term follow-up of anti-VEGF treatment. SEVEN-UP and CATT were among the first studies to describe the long-term outcomes with either monthly or PRN dosing.^[13,14]^ Of the TAE trials, the longest to date was by Mrejen et al over a six-year period with 185 patients and a retention rate of 62.9%.^[12]^ Their study showed an improvement or maintenance in visual acuity with an average of 8.3 injections per year. They demonstrated that a greater number of injections was an independent predictor of better visual outcome. Other studies have compared TAE to PRN revealing a worse visual outcome with a smaller number of injections with the PRN group.^[15,16]^ Specifically, Calvo et al showed that over a three-year follow-up period, 42.4% in the TAE dosing group versus 24.1% in the PRN dosing group gained at least three lines of vision. Over the study period, the TAE group was treated with an average of 20.31 injections, while the PRN group was treated with an average of 18.41.^[15]^ TAE is a practical option to reducing the number of injections and office visits as compared to a monthly and PRN regimen.

Our current study reports a seven-year follow-up period of the long-term outcomes as measured by visual acuity and OCT imaging of the treatment-naïve NV-AMD patients using a TAE model.

##  METHODS

The Institutional Review Board of Northwestern University Feinberg School of Medicine approved this retrospective cohort study at a large urban tertiary medical center. Study data was obtained through the Northwestern Medicine Enterprise Data Warehouse (NMEDW) and through direct chart review. Our study population consisted of treatment-naïve patients of four retina specialists receiving intravitreal anti-VEGF with the diagnosis of neovascular AMD (ICD-9 code 362.52) from March 2008 to October 2015. Other inclusion criteria were: age more than 50 years, visual acuity of hand motion (HM) or better at baseline, and a follow-up duration of at least one year. All four physicians used a TAE protocol consisting of initial intensive monthly anti-VEGF injections until there was no evidence of exudation on OCT followed by extension of treatment interval by two weeks up until 12 weeks. If there was a mild recurrence of subretinal fluid (SRF), intraretinal fluid (IRF), or a new macular hemorrhage, then the interval was reduced by one–two weeks until the macula was dry or hemorrhage stabilized. In the case of more severe recurrences, monthly treatment was reinitiated.^[10]^ The interval was not increased in the presence of persistent but stable fluid, however, if a pigment epithelial detachment (PED) persisted in the absence of SRF or IRF, then the interval was extended. Patient demographics, type of anti-VEGF agent used (bevacizumab, ranibizumab, or aflibercept), number and frequency of injections, best-corrected visual acuities (BCVA), and intraocular pressure (IOP) were obtained at each office visit from the EDW database. In this article, single-agent monotherapy is defined as treatment with only one type of anti-VEGF agent over the entire treatment course, while multi-agent therapy is defined as treatment with multiple types of anti-VEGF agents but not during the same office visit.

OCT images of the affected eye were obtained from direct chart review at baseline and at the last follow-up visit. OCT images of each affected eye at baseline and the last follow-up visit were directly reviewed for the presence or absence of SRF and IRF. BCVA and IOP were extracted for each affected eye at the start of treatment and at time-points of six months, one year, and every year thereafter until the last office visit. Measurements from the office visit whose date was closest to the specific time-point were selected but was required to be within three months of the specific time-point to be included. Visual acuity values were converted from Snellen to logMAR to allow the paired *t*-test comparisons. Visual acuity values were also categorized as 20/50 or better, between 20/50 and 20/200, and 20/200 or worse for further interpretation. The number and types of injections were also tallied for each affected eye.

Subsequent numerical and statistical analyses were performed in Microsoft Excel 2016 (Microsoft Corporation, Redmond, WA) and GraphPad Prism 7 (GraphPad Software, San Diego, CA). The paired Student *T*-test was performed to compare visual acuities and intraocular pressure at the first and last office visits. Pearson's correlation coefficient was calculated to test for the linearity of changes in visual acuity over time. Statistical analysis was also performed for visual acuity categories using contingency tables with Fisher's exact test and the OCT data was analyzed with McNemar's test. Subgroup analysis was conducted on eyes treated with single-agent monotherapy to examine the visual acuity and OCT outcomes for each drug.

##  RESULTS

In total, 224 treatment-naïve eyes of 202 patients were analyzed with an average follow-up period of 3.4 years (range, 1.0–7.6 years). The majority (80%) of patients in this study were between 70 and 89 years of age at initial presentation. Of the 224 eyes, 137 (61.2%) were treated with only one type of anti-VEGF agent: ranibizumab (71, 51.8%), aflibercept (47, 34.3%), or bevacizumab (19, 13.9%). Visual acuity at baseline was 20/100 in Snellen and did not differ significantly between the treatment groups (F = 1.33, *P* = 0.27). Most eyes had either SRF (70%) or IRF (47%) present on OCT imaging at baseline (Table 1).

**Table 1:** Patient baseline characteristics


	**Monotherapy (** ***N*** ** = 137)**			**Multi-drug therapy (** ***N*** ** = 87)**	**Total (** ***N*** ** = 224)**
	Bevacizumab (*N* = 19)	Ranibizumab (*N* = 71)	Aflibercept (*N* = 47)	
**Eye – no (%)**						
	OD	7(37)	33 (47)	21 (45)	48 (55)	109 (49)
	OS	12(63)	38 (54)	26 (55)	39 (45)	115 (51)
**Gender – no (%)**						
	F	16(84)	49 (69)	38 (81)	58 (67)	161 (72)
	M	3(16)	22 (31)	9 (19)	29 (33)	63 (28)
**Race – no (%)**						
	Caucasian	12(63)	52 (73)	40 (85)	72 (83)	176 (79)
	African American	2(11)	4 (6)	0	5 (6)	11 (5)
	Hispanic	3(16)	1 (1)	0	2 (2)	6 (3)
	Other/Unknown	2(11)	14 (20)	7 (15)	8 (9)	31 (14)
**Age**						
	Mean	78.1 ± 12.6	82.1 ± 6.5	83.3 ± 6.4	78.2 ± 8.7	80.5 ± 8.3
	50–69 -no. (%)	4 (21)	2 (3)	2 (4)	13 (15)	21 (9)
	70–89 -no. (%)	12 (63)	62 (87)	40 (85)	66 (76)	180 (80)
	90+ -no. (%)	3 (16)	7 (10)	5 (11)	8 (9)	23 (10)
**Visual Acuity (Snellen)**						
	Mean	20/94	20/124	20/91	20/89	20/100
	20/50 or Better - no. (%)	7 (37)	19 (27)	20 (43)	32 (37)	78 (35)
	Between 20/50 and 20/200 - no. (%)	7 (37)	29 (41)	17 (36)	33 (38)	86 (38)
	Worse than 20/200 - no. (%)	5 (26)	23 (32)	10 (21)	22 (25)	60 (27)
**OCT Findings**						
	SRF-no. (%)	14 (74)	42 (59)	30 (64)	73 (84)	159 (71)
	IRF-no. (%)	8 (42)	43 (61)	27 (57)	28 (32)	106 (47)
OCT, optical coherence tomography; SRF, subretinal fluid; IRF, intraretinal fluid						

**Table 2:** OCT* characteristics of single-agent vs multi-agent anti-VEGF therapy


**Anti-VEGF Drug for Single-agent Monotherapy**	**# SRF + Pre-treatment**	**# SRF + Post-treatment**	**** ***P*** **-value**	**# IRF + Pre-treatment**	**# IRF + Post-treatment**	**** ***P*** **-value**
**Bevacizumab (** ***N*** ** = 19)**	14	2	*P* < 0.01	8	2	*P* = 0.077
**Ranibizumab (** ***N*** ** = 71)**	42	8	*P* < 0.001	43	16	*P* < 0.001
**Aflibercept (** ***N*** ** = 47)**	30	1	*P* < 0.001	27	10	*P* < 0.001
**Multi-agent Therapy** **(** ***N*** ** = 87)**	73	24	*P* < 0.001	28	14	*P* < 0.001
**Total Cohort** **(** ***N*** ** = 224)**	159	35	*P* < 0.001	106	42	*P* < 0.001
OCT, optical coherence tomography; SRF, subretinal fluid; IRF, intraretinal fluid; VEGF, vascular endothelial growth factor						

**Figure 1:**
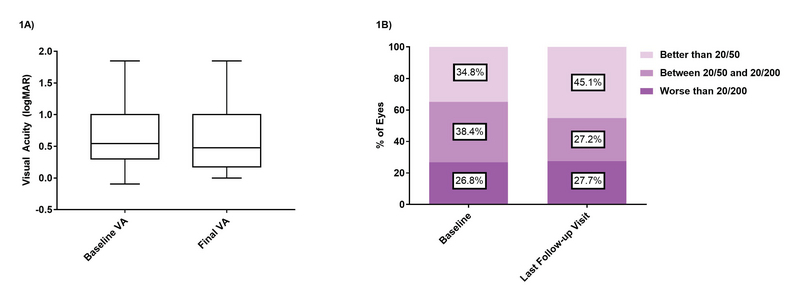
Comparison of mean visual acuity between baseline and last visit. Figure 1A shows boxplot comparisons between baseline and final visual acuities. Figure 1B shows the percentage of patients in each visual acuity category by the last follow-up visit compared to baseline.

The average visual acuity at baseline of 0.698 logMAR (20/100 Snellen equivalent) remained stable at the final follow-up visit at 0.666 logMAR (20/93 Snellen) (*P* = 0.30; Figure 1a). A significant portion of eyes (40%) maintained their visual acuities within two Snellen lines, while 34% of eyes gained more than two lines and 25% lost more than two lines. The percentage of eyes with visual acuities of 20/50 or better increased significantly from 34.8% at baseline to 45.1% by the last follow-up visit (Fischer's exact test, *P* = 0.037; Figure 1b). There was no significant difference between baseline IOP (14.8 ± 3.3) and IOP at the last follow-up visit (15.2 ± 3.6) (*P* = 0.052).

**Figure 2:**
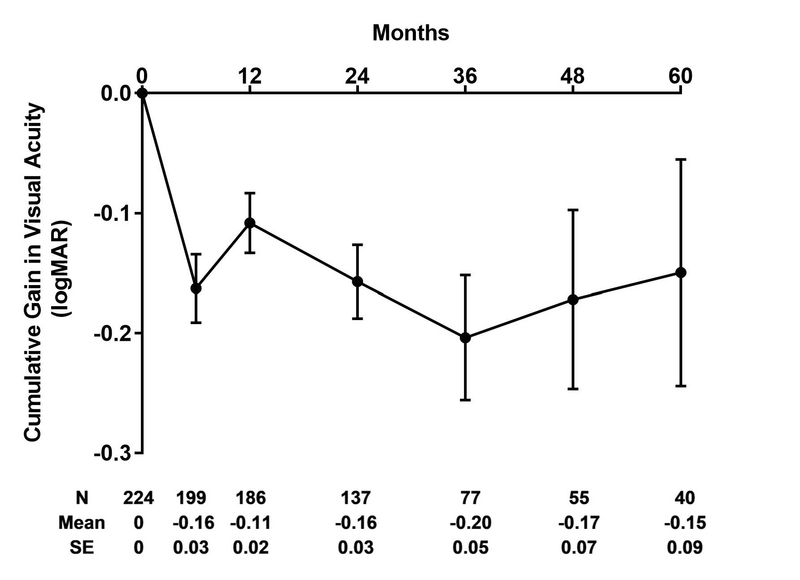
Cumulative gain in logMAR over treatment course (Mean ± SE). Visual acuities recorded during patients' treatment visits were compared with the visual acuity at baseline. Only those patients actively continuing to receive injections were included in this figure; patients who discontinued injections after a specific time were not included in subsequent time points in this graph.

**Figure 3:**
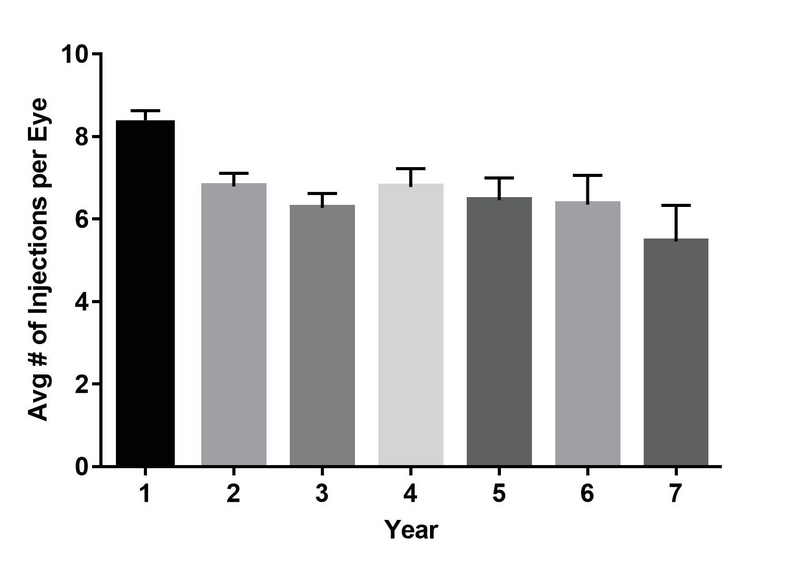
Number of injections over time. The figure shows the average annual number of injections administered over time.

**Figure 4:**
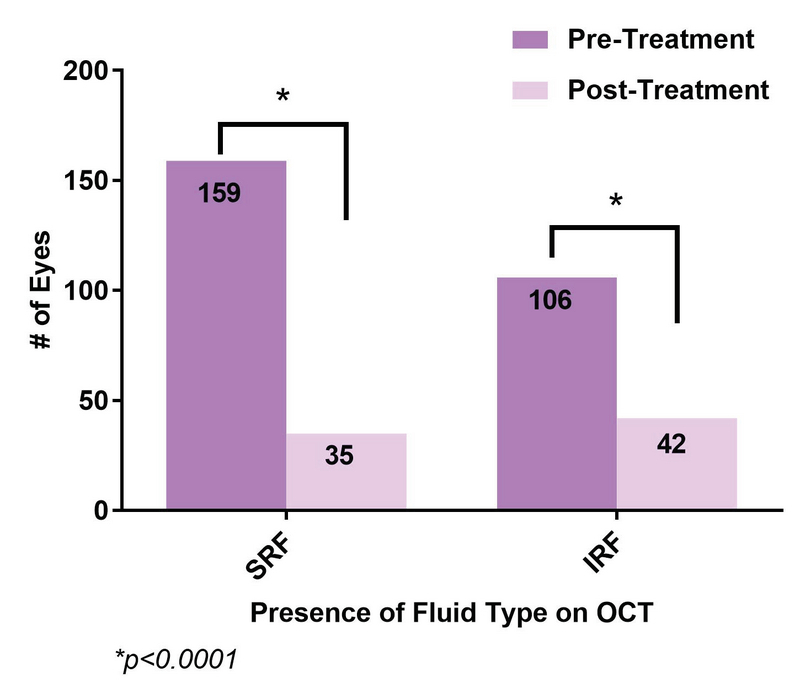
Comparison of OCT finding between baseline and last visit. The figure shows the number of eyes with the presence of SRF and IRF pre- and post-treatment.

Visual acuities of patients receiving ongoing injections recorded at six months, one year, and annually thereafter showed an overall steady gain that peaks near the end of the third year, with slight reductions thereafter (Figure 2). The smaller sample size in these groups impedes any individual subgroup analysis of the treatment type.

The baseline visual acuity at initial presentation was tested against the change in visual acuity along with demographic factors of sex and age as possible predictors of patient's response to treatment. Baseline visual acuities exhibited a weak linear correlation with the cumulative change in visual acuities at all time-points (Pearson's coefficient r averaged over timepoints = –0.45, *P* < 0.05). Age was not found to be linearly correlated with the change in visual acuity at the last follow-up (Pearson's r = 0.13, p = 0.33). Similarly, patients' sex and also race (Caucasian vs non-Caucasian) were not correlated with the treatment response (t = –1.13, *P* = 0.26 and t = –0.6, *P* = 0.55, respectively).

Over the course of the study, 224 eyes received an average of 20.3 ± 14.7 injections (range, 2–95) during a mean of 3.4 years of follow-up (range, 1.0–7.6), for a total of 4,543 injections. Of the 224 eyes, 137 (61.2%) were treated with a single agent for the duration of their treatment [71 (51.8%) with ranibizumab, 47 (34.3%) with aflibercept, and 19 (13.9%) with bevacizumab] while 87 (38.8%) eyes were treated with more than one agent type. For those patients receiving single-agent therapy, the number of total injections did not differ significantly based on the agent used (14.8 for bevacizumab vs 14.7 for ranibizumab vs 13.0 for aflibercept, *P* = 0.54) over the course of treatment, although the average duration of treatment in weeks varied significantly (35.5 for bevacizumab vs 28.6 for ranibizumab vs 21.7 for aflibercept, *P* = 0.014). The number of injections that patients received differed over time (Figure 3). Eyes in the first year of treatment received an average of 8.4 injections that decreased on average by 0.3 injections per year to 5.5 injections by the seventh year (R${}^{2}$ = 0.68). Moreover, eyes that gained more than two lines received significantly more injections with an average number of 24.1 ± 15.3 injections, while eyes that maintained within two lines or lost more than two lines received 18.1 ± 13.3 injections and 18.6 ± 15.2 injections, respectively (*P* < 0.05). It was also found that eyes with visual acuities of 20/200 or better at last follow-up tended to receive treatment over a longer period (average 3.1 years vs 2.2 years, *P* < 0.01) and received a greater number of injections (average 23 vs 14, *P* < 0.001) compared to eyes with visual acuities of 20/200 or worse at last follow-up.

In addition to visual acuity analysis, OCT images were compared at baseline and at the last follow-up visit. Out of the 224 eyes, 159 eyes had SRF at baseline compared with 35 eyes by the date of last follow-up (*P* < 0.0001; Figure 4). Similarly, 106 eyes had IRF at baseline compared with 42 eyes by the date of last follow-up (*P* < 0.0001). When aggregated, 208 eyes had some type of fluid at baseline, compared with 69 eyes by the date of last follow-up (*P* <0.0001). Subset analysis of eyes receiving single-agent anti-VEGF therapy did not reveal any differences in OCT outcomes and mirrored the trends seen in the overall group. All treatment groups showed a statistically significant decrease in the presence of fluid over the course of the treatment except for the presence of IRF in the bevacizumab group (Table 2), although this may be attributed to the smaller sample size of this subgroup (*n* = 19).

##  DISCUSSION

We report up to a seven-year (average, 3.4 years) follow-up period of treatment-naïve NV-AMD patients undergoing anti-VEGF therapy using a TAE model. All four investigators in our study used the consensus recommendations of the TAE regimen following monthly injections until the macula was dry based on OCT, then extending the interval between treatments by two weeks to a maximum of twelve weeks. If fluid recurred, then the interval would be shortened. Using this approach, the patients' treatment is individually tailored to its response. The TAE regimen offers an alternate and preferred treatment practice due to reduced burden for office visits compared to monthly and OCT-guided dosing regimens.

Prior to the development of TAE regimen, long-term outcomes of monthly and PRN anti-VEGF treatments have been described in several other studies, most notably in the SEVEN-UP and 5-year CATT study.^[13,14]^ The SEVEN-UP study reported on ranibizumab-treated patients after an average of 7.3 years from the time of first injection with patients receiving monthly injections in the first two years followed by PRN treatment over the subsequent years. Patients received an average of 6.8 total injections over a mean 3.4 year interval. The subgroup that received more frequent injections yielded a better result in visual acuity gains. In their study, 23% attained a visual acuity of 20/40 or better whereas 37% were 20/200 or worse. There was an overall mean decline of 8.2 letters over the course of follow-up.^[14]^

In the five-year CATT study, patients were followed an average of 5.5 years from the time of first injection. Ranibizumab- or bevacizumab-treated patients were stratified into monthly or PRN arms in the first year with the monthly arm stratified again into monthly or PRN treatment in the second year. In the subsequent three years, a variety of treatment drug combinations and regimens were used with patients receiving an average of 15.4 injections over three years. In their study, 49.6% attained visual acuity of 20/40 or better whereas 20% were 20/200 or worse. There was a mean overall decline of 3.3 letters.^[13]^

Since the initial description of the TAE regimen, several studies have reproduced results favoring maintenance or improvement of BCVA similar to monthly dosing while reducing injection frequency and treatment burden. Our study is comparable to others that describe outcomes using a TAE regimen.^[18,19,20,21,22,23]^ Similar to our study, BCVA in these studies was either maintained or improved throughout treatment with 30–34% of patients on average improving by 2–3 lines and 94–97.5% patients losing less than 2–3 lines. The number of injections in the first year averaged a total of 7.6–8.6, which is comparable to our mean of 8.4. Most of these studies, however, only reported on outcomes over a two-year follow-up while our study looks at outcomes over a longer treatment period. Of note, in our study, the average number of injections decreased to 5.5 during the seventh year while maintaining BCVA.

A study by Mrejen et al with a longer follow-up period of six years (average 3.5 years) compared to the aforementioned studies^[18,19,20,21,22,23]^ demonstrated similar favorable results.^[10,11,12]^ In their study, BCVA peaked at 18 months with a steady decline afterward. On average, patients received 28.5 injections over the study period with 8.3 injections per year and a mean interval of 6.6 weeks between injections. The majority of their patients (64.3%) were treated with injection of a single agent of which 59% of them were ranibizumab alone, 4.3% were bevacizumab alone, and 1% was aflibercept alone. Their multivariant analysis showed a greater number of injections as an independent predictor of better visual outcomes. On the other hand, older age of starting injections, hypertension, and anticoagulation were correlated with poorer visual outcomes.

In the current study, we utilized a TAE approach in which patients were followed for an average of 3.4 years (range, 1.0–7.6) receiving an injection regimen with an average of 20.3 ± 14.7 total injections with 8.4 injections in the first year and 5.5 injections by the seventh year of follow-up. The majority of patients (61.1%) were treated with a single anti-VEGF agent for the duration of their treatment, of which 51.8% were ranibizumab alone, 34.3% were aflibercept alone, and 13.9% were bevacizumab alone. Having more single-agent data analysis allows us to validate similarities of BCVA outcomes regardless of the drug type. BCVA peaked after three years of treatment with a slow decline thereafter. Baseline visual acuity was weakly shown to be the only significant predictor of change in visual acuity. There were no significant differences between drug type and visual acuity effect, number of injections needed, or OCT outcomes.

Eyes with a final visual acuity of 20/50 or better increased from 34.8% at the beginning of the study to 45.1% at the latest follow-up (*P* = 0.037), while eyes with 20/200 or worse remained stable at 26.8% at baseline compared to 27.7% at last follow-up (*p* = 0.92). In contrast, in the five-year CATT study, eyes with 20/200 or worse increased significantly from 6% at baseline to 20% at the last follow-up^[13]^ even though in our study there were more patients with baseline vision of 20/200 or worse. At the end of the SEVEN-UP study, 37% of patients were reported to have visual acuity of 20/200 or worse. Overall, 74% of patients in our study maintained or gained at least two Snellen lines of visual acuity, compared with the SEVEN-UP trial where only 55% of eyes maintained or improved their vision.^[14]^ At the end of our study, eyes that improved by at least two lines received an average of 24 injections, while all other eyes received an average of 18 injections. Similarly, in the SEVEN-UP study, eyes receiving a greater number of injections (11 vs 6.8) gained 3.9 letters overall and were more likely to show improvement in vision.^[14]^

The better visual outcomes in our population compared to the five-year CATT study may be explained by the OCT analysis. At the last follow-up visit, 16% of eyes in our study had SRF and 19% of eyes had IRF. Those without SRF or IRF had either a PED or were without any fluid. In comparison, at the end of five years in the CATT study, 38% had SRF and 61% had IRF.^[13]^ Eyes with residual IRF yield worse visual outcomes compared with eyes with residual SRF or absence of fluid.^[16]^ More frequent treatments may have an impact on the amount of fluid on OCT to allow for maintenance or gain in visual acuity. However, other factors such as geographic atrophy also contribute to the final visual acuity. Though not studied in our population, the CATT study demonstrated that 24% of eyes with monthly dosing showed geographic atrophy compared to 15% in the PRN group. Similarly, in the IVAN trial, 34% versus 26% showed progressive atrophy in the monthly versus PRN groups, respectively.^[8,13,17]^

Our study demonstrates that a TAE model allows for a frequent albeit lower treatment burden as compared to monthly dosing with reduction in fluid on OCT. This may theoretically lower the rate of geographic atrophy while maintaining similar gains in visual potential compared with a monthly dosing regimen.

There are several limitations in our study most notably its retrospective nature. With data being compiled via electronic database, records may be incomplete and there may be innate errors in how the data was recorded. Patients began treatment at different times between 2008 and 2015 and there may be differences both in medical technology and in practice patterns amongst providers. There is no monthly regimen treatment arm to compare its efficacy with our TAE model. Due to the method of data collection, there were fewer patients with more than four to five years of treatment available for analysis thereby limiting sample size and comparisons across different anti-VEGF agents. Some patients have undergone cataract surgery during treatment period, which can confound BCVA amongst patients. Furthermore, more in-depth studies are needed to analyze the impact of residual fluid type on visual acuity.

In conclusion, our retrospective uncontrolled review of a large urban cohort of NV-AMD reveals favorable long-term visual and anatomic results of anti-VEGF therapy using a TAE regimen. Our study demonstrates that visual acuity seems to improve with more frequent injections over a longer period of time. The majority of patients (74%) maintained or improved vision with 45% of patients achieving VA of 20/50 or better at their last follow-up over a seven-year period. Our study supports the use of a TAE treatment paradigm to reduce both office visits and treatment burden while still achieving positive functional and anatomic results.

##  Financial Support and Sponsorship

This study was supported in part by an unrestricted grant from Research to Prevent Blindness and by the Northwestern Medicine Enterprise Data Warehouse. The sponsor or funding organization had no role in the design or conduct of this research.

##  Conflicts of Interest

There are no conflicts of interest.
